# Berberine Reduces Aβ_42_ Deposition and Tau Hyperphosphorylation *via* Ameliorating Endoplasmic Reticulum Stress

**DOI:** 10.3389/fphar.2021.640758

**Published:** 2021-07-19

**Authors:** Yue Wu, Qingjie Chen, Bing Wen, Ninghua Wu, Benhong He, Juan Chen

**Affiliations:** ^1^ Department of Biochemistry and Molecular Biology, School of Basic Medicine and the Collaborative Innovation Center for Brain Science, Tongji Medical College, Huazhong University of Science and Technology, Wuhan, China; ^2^ Hubei Key Laboratory of Diabetes and Angiopathy, Hubei University of Science and Technology, Xianning, China; ^3^ Basic Medical College, Hubei University of Science and Technology, Xianning, China; ^4^ Department of Cardiovascular Medicine, Lichuan People’s Hospital, Lichuan, China

**Keywords:** berberine, Alzheimer’s disease, endoplasmic reticulum stress, Aβ_42_ production, tau hyperphosphorylation

## Abstract

Alzheimer’s disease (AD) is tightly related to endoplasmic reticulum stress (ER stress), which aggravates two dominant pathological manifestations of AD: senile plaques and neurofibrillary tangles. Berberine is widely applied in the clinical treatment of many diseases and is reported to have anti-AD effects. In the present study, berberine was shown to ameliorate ER stress and cognitive impairment in APP/PS1 mice. We found ER stress plays a role as a central hub for signal transduction, which was evidenced by the hyperactivation of glycogen synthase kinase 3β (GSK3β) to phosphorylate tau and the activation of PRKR-like endoplasmic reticulum kinase (PERK) subsequently to phosphorylate eukaryotic translation initiation factor-2 α (eIF2α). Also, eIF2α has regulated the expression of beta-site APP cleaving enzyme-1 (BACE1), which cleaves APP into pro-oligomerized amyloid beta 42 (Aβ_42_), the main component of senile plaques, proven by using siRNA targeting at eIF2α. Mechanically, berberine can reduce GSK3β activity, contributing to the downregulation of tau phosphorylation. Berberine also suppressed Aβ_42_ production *via* inhibiting the PERK/eIF2α/BACE1 signaling pathway. Taken together, these findings indicated that berberine had the potential to ameliorate two major pathological manifestations of AD mainly by suppressing ER stress. Our work provided knowledge on the pharmacological intervention of AD and the possible targets for future drug development.

## Introduction

Alzheimer’s disease (AD) is the most common neurodegenerative disease that induces progressive dementia and worsens life quality, causing heavy burden to the family as well as society (Alzheimer’s, 2016). The dominant pathological manifestations associated with AD include extracellular senile plaques and intracellular neurofibrillary tangles (NFTs) (Long and Holtzman, 2019). The senile plaques are considered as deposits of aggregated amyloid beta (Aβ), which in turn is produced by subsequent cleavage of amyloid precursor protein (APP) by β-secretases and γ-secretases (Long and Holtzman, 2019). NFTs comprise hyperphosphorylated tau protein, and the tau protein is phosphorylated by several kinases, including glycogen synthase kinase 3b (GSK3β), forming matured and stable microtubules (Tiwari et al., 2019). The production of senile plaques and hyperphosphorylated tau is triggered by different signals, wherein the recent studies have indicated several overlapping manifestations between these two (Chauhan and Chauhan, 2006; Long and Holtzman, 2019), which also includes endoplasmic reticulum stress (ER stress) (Salminen et al., 2009; Guix et al., 2019).

Increasing studies have revealed that ER stress is observed in the postmortem brains of patients as well as animal models with AD (Katayama et al., 2004; Hoozemans et al., 2005; Hoozemans et al., 2009; Mota et al., 2015). The accumulation of unfolded proteins could disrupt the homeostasis of the ER, inducing ER stress (Yoshida, 2007). A variety of signaling proteins are activated under ER stress; therefore, it is regarded as a critical process in the etiology of AD (Salminen et al., 2009; Huang et al., 2015). But the precise mechanisms of how ER stress promotes the production of senile plaques and hyperphosphorylated tau are still not fully elucidated. Relieving ER stress might affect the delay of progression and prevent the deterioration of AD. Meanwhile, there are few studies that reported amelioration of cognitive defects in AD mice by inhibiting the downstream sensors of ER stress (Axten et al., 2012; Ma et al., 2013; Sidrauski et al., 2013). So, it is necessary to find if there is any solution to the key signaling hub in patients with AD.

Berberine (BBR) is a natural isoquinoline alkaloid that is purified from the traditional Chinese medicine *Coptis chinensis*, and it has been widely used as a commercialized drug for treating various diseases. The neuroprotective role of BBR has been discovered recently (Ji and Shen, 2011). Several evidences have demonstrated that BBR can alleviate cognitive impairment through varied effects, including antioxidant, anti-inflammatory, and alleviating hyperphosphorylation of tau as well as reducing Aβ production (Ji and Shen, 2011; He et al., 2017; Cai et al., 2018; Chen et al., 2020). Our previous studies have confirmed that BBR has a good curative effect in relieving the cognitive impairment caused by diabetes (Chen et al., 2017; Wang et al., 2018; Wang et al., 2019). However, the underlying mechanism of neuroprotective function of BBR still remains to be unclear till date. Meanwhile, studies have revealed that BBR inhibits ER stress in several diseases, except AD (Wang et al., 2010; Li et al., 2018; Liu et al., 2019), and whether BBR affects ER stress in AD has not been investigated.

Hence, in this study, the APP/PS1 transgenic mice and mouse hippocampus neuron cell line HT22 with APP stably expression were utilized to investigate AD-related pathological changes both *in vivo* and *in vitro*, and explore the detailed underlying mechanism associated with the protective effects of BBR. Our results showed that BBR can alleviate ER stress in the AD model both *in vivo* and *in vitro* mainly by inhibiting tau hyperphosphorylation and Aβ_42_ production and deposition.

## Materials and Methods

### Animals and Treatments

Six-month-old male APP/PS1 transgenic mice: Mo/HuAPP Swedish mutations (K595N/M596L) +Hu PS1 delta E9 and age/sex-matched wild-type C57BL/6 mice were purchased from Beijing Vital River Laboratory Animal Technology Co. Ltd. The mice were housed in the Experimental Animal Center of Tongji Medical College in specific pathogen-free environment. All experimental procedures were approved by the Animal Care and Use Committee of the Huazhong University of Science and Technology (No. 2019S2126) and were performed in compliance with the National Institutes of Health Guidelines on the ethical use of animals. The mice were housed three to five per cage in a room maintained at consistent ambient temperature (22 ± 2°C) and humidity (50 ± 5%), with an alternating 12-h light–dark cycle. Mice were allowed free access to food and water *ad libitum*. The APP/PS1 mice and controls were randomly assigned into four groups, with *n* = 15 mice in each group: wild-type (WT) group, WT+BBR group, APP/PS1 group, and APP/PS1+BBR group.

The dose of BBR for mouse is 260 mg/kg and was added into the diet. The WT+BBR group and APP/PS1+BBR group received BBR diet for 3 months, and other groups were given standard diet. Behavioral testing was performed prior to one week of sacrifice.

### Morris Water Maze Test

The cognitive function of the mouse was assessed using a Morris water maze (MWM) test as reported previously (Shi et al., 2018). Briefly, the water maze was divided into four equal quadrants. A hidden square platform was submerged below 1 cm water level and placed in the third quadrant of the pool. The mice were allowed for 2 days to adapt to the pool environment. The training trial was then conducted for six consecutive days. The escape latency, distance, and time were recorded by an automated video tracking system and software (NoldusEtho Vision 2.3.19, Netherlands). The behaviors of the mice were tracked using EthoVision 3.0.

### Cell Cultures and Treatments

The mouse hippocampal neuron cell line HT22 was cultured in Dulbecco’s modified eagle’s medium containing 10% fetal bovine serum (Gibco, United States) in a humified incubator under 5% CO_2_ at 37°C. HT22/APP (HT22 cells stably transfected with Swedish mutant form of APP) cell line was constructed by transfecting APPswe plasmid into HT22 cells using Lipofectamine 2000 and selecting the single-cell clones with G418. The HT22/APP cells were pretreated with or without BBR (5 μM, Solarbio), 4-phenylbutyrate (PBA, 1 mM, Aladin), and SB216763 (the GSK3β inhibitor) (10 μM, Abcam) for 1 h, and then stimulated with thapsigargin (TG, 1 μM, Aladin) for 8 h. siRNA-eukaryotic translation initiation factor-2 α (eIF2α) (SANTA CRUZ, sc-78173) was transfected with Lipofectamine 2000 reagent for 24 h to silence the expression of eIF2α.

### Western Blotting

Western blotting was conducted as reported previously (Shi et al., 2018). Briefly, the cells or hippocampal tissues were lysed by RIPA lysis buffer with a protease inhibitor PMSF followed by the addition of phosphatase inhibitor cocktail and incubation on ice for 15 min with vortex for 30 s for 5 min. After centrifugation at 12,000 rpm for 15 min at 4°C, the supernatant was collected and quantified *via* BCA Assay Kit (Thermo, #23225). The proteins were mixed with 5X loading buffer and boiled for 10 min at 100°C. Next, the proteins were separated in SDS-PAGE gel and then transferred on to the PVDF membrane (Millipore, CA). The membrane was blocked with 5% bovine serum albumin (BSA) in TBS/Tween20 (1%) for 1 h at room temperature (RT), and then incubated overnight with primary antibodies at 4°C. Horseradish peroxidase–conjugated secondary antibody was used to visualize the targeted band by Bio-Rad GelDoc^TM^ XR and ChemiDoc^TM^ XRS System. The primary antibodies used were as follows: β-actin (AB clonal, AC028, 1:3000), tau/ps404 (Abcam, ab30666, 1:1,000), tau/ps202 (Abcam, ab108387, 1:1,000), binding-immunoglobulin protein (Bip) (AB clonal, A0241, 1:1,000), p-GSK3β Y216 (AB clonal, AP0261, 1:1,000), GSK3β (CST, #12456,1:1,000); eIF2α (Abcam, ab169528, 1:1,000), p‐eIF2α Ser51 (CST, #3398, 1:1,000), PRKR-like endoplasmic reticulum kinase (PERK) (Abcam, ab65142, 1:1,000), p-PERK T980 (CST,#3179, 1:1,000), APP (AB clonal, A16265, 1:1,000), APP-C-terminal fragment 99 (CTF99) (AB clonal, A11019, 1:1,000), and beta-site APP cleaving enzyme-1 (BACE1) (AB clonal, A5266, 1:1,000).

### Immunofluorescence Staining and Immunohistochemical Staining

Cells were fixed with 4% paraformaldehyde for 10 min at RT and then subsequently were permeated by 0.1% Triton X-100 for 10 min at RT. After that, 5% BSA was used to block nonspecific signals for 30 min. The cells were then incubated with primary antibody at 1/200 dilution for overnight at 4°C. Paraffin sections were deparaffinized, rehydrated, and antigen retrieved for immunofluorescence staining. The sections were blocked by 5% BSA followed by overnight incubation in primary antibody at 4°C. The anti-rabbit fluorescence secondary antibody was then incubated at 1/1,000 dilution for 1 h at RT by avoiding light. Finally, 4’,6-diamidino-2-phenylindol was used to stain the nuclei. The images were acquired at ×40 magnification by using Olympus microscopy.

The sections were pretreated and the slips were incubated with Aβ_42_ primary antibody (CST,#24090, 1:200) and tau ps 404 antibody for overnight at 4°C. On the next day, the slips were incubated with secondary antibody for 2 h at RT, and 3,3’-diaminobenzidine (DAB) was added to show positive signal. The section was counterstained with Mayer’s hematoxylin. Finally, the DAB-stained slips were visualized under Olympus AX-70 microscope equipped with a motorized stage.

### Transmission Electron Microscope

TEM (Hitachi, Japan) was used to observe the ultrastructure changes. In advance, the tissue blocks were fixed with glutaraldehyde. The tissues were then embedded and sliced with ultramicrotome after rinsing and dehydrating in ethanol. TEM was used for observing the morphology of the ER and obtaining pictures.

### Enzyme-Linked Immunosorbent Assay

Aβ_42_ levels were quantified by ELISA kit (ElabScience) according to the manufacturer’s instructions. After incubation, the optical density values were detected at 450 nm using a spectrophotometer (Synergy2, United States) after a period of reaction. The Aβ_42_ contents were calculated according to the standard curve drawn and by using the reference substance in the same system.

### Statistical Analysis

Data were expressed as means ± standard error of mean (SEM) and analyzed by Graph Pad Prism 5.0 software. One-way ANOVA following Tukey’s *post hoc* test was used to assess significant differences among the groups. *p*-values of <0.05 were considered to be statistically significant.

## Results

### Berberine Alleviates Endoplasmic Reticulum Stress in Amyloid Precursor Protein/PS1 Mice

**FIGURE 1:**
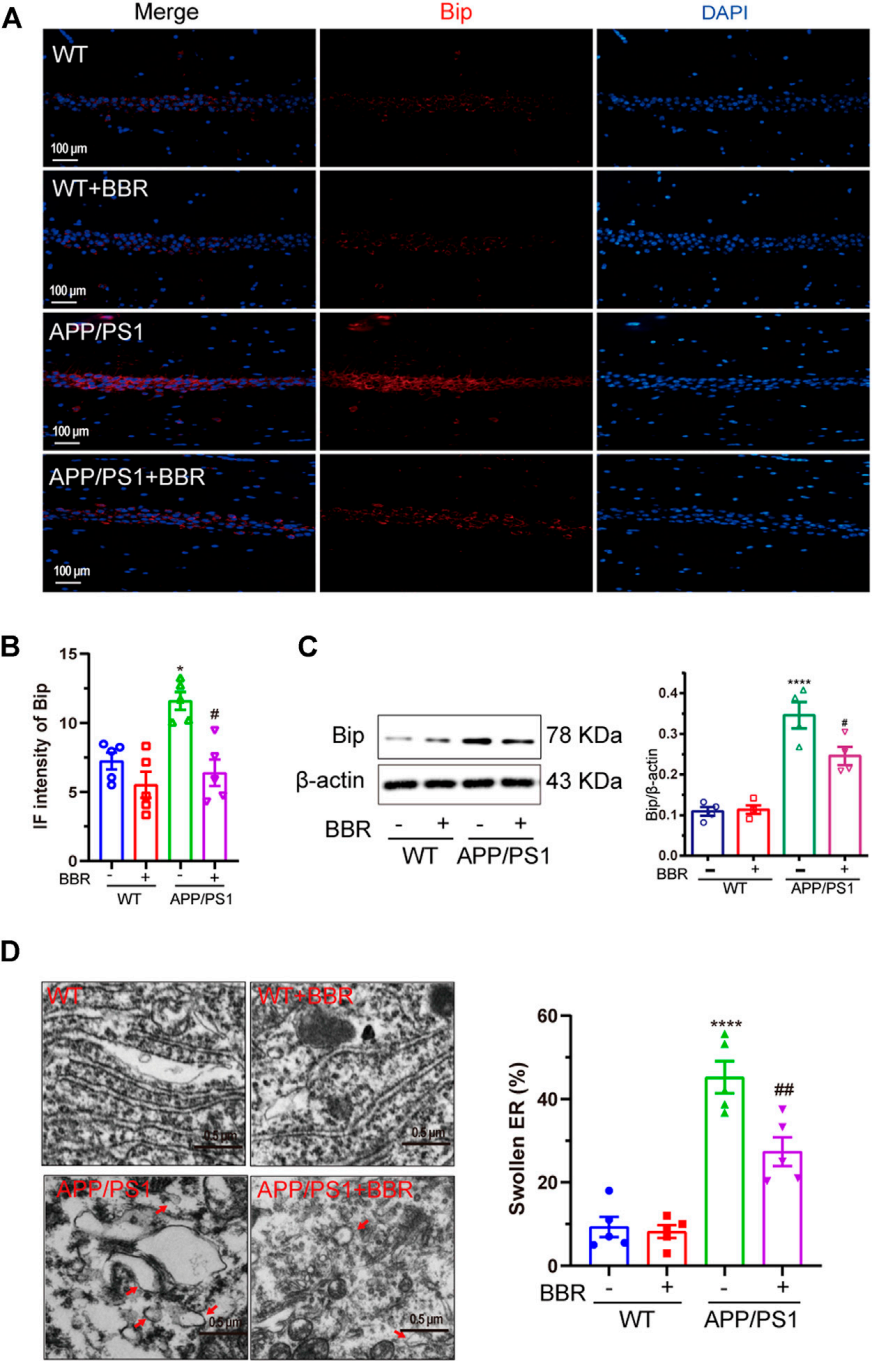
Berberine alleviates ER stress in APP/PS1 mice. **(A)** Representative immunofluorescence images of Bip in the CA1 region of the hippocampal tissue sections. Scale bar is 100 μm. **(B)** Quantification of IF intensity of Bip. **(C)** Western blot detection of Bip expression in the hippocampal tissues of the mice and quantitative analysis. **(D)** Representative TEM images of the endoplasmic reticulum structure in the CA1 region of hippocampal tissues and quantitative analysis. Scale bar is 0.5 μm. Data were presented as means ± SEM. ****p < 0.0001 vs. WT; ^#^ *p* < 0.05, ^##^ *p* < 0.01 vs. APP/PS1 without BBR.

It is widely accepted that Bip is a distinguished marker of ER stress occurrence (Lee, 2005). Immunofluorescence (Figures 1A,B) and Western blotting (Figure 1C) results revealed that Bip was significantly upregulated in the CA1 region of the hippocampus of APP/PS1 mice. It is quite interesting that the upregulated Bip expression in APP/PS1 mice was almost completely reversed after treatment with BBR. Also, TEM was used to observe the ultrastructure of hippocampal tissue. As shown in Figure 1D, the ER lumen was compacted and elongated in WT mice and WT mice that underwent BBR treatment. However, in APP/PS1 mice, the ER morphology was apparently swollen, losing normal morphology. With BBR treatment, the morphology of the ER returned to normal in APP/PS1 mice, which further supported that BBR treatment could alleviate ER stress.

### Berberine Ameliorates Cognitive Impairment in Amyloid Precursor Protein/PS1 Mice

**FIGURE 2:**
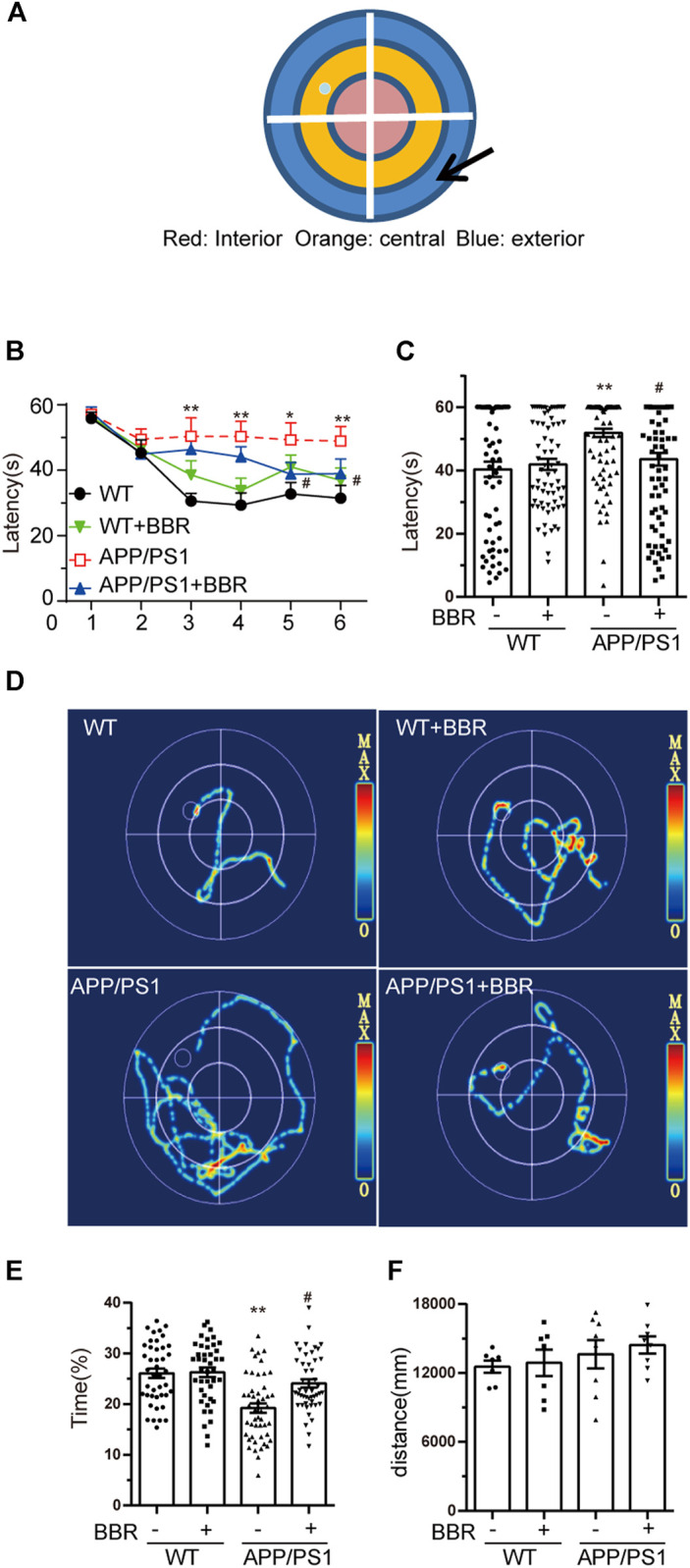
Berberine improves the cognitive ability of APP/PS1 mice. **(A)** MWM schematic. **(B,C)** Escape latency analysis in the navigation training trials. **(D)** The trajectory of mice in MWM. **(E)** The percent of time spent in the target quadrant during the space exploration experiment. **(F)** Analysis of swimming distances during trials. Data were presented as means ± SEM. *p < 0.05, **p < 0.01 vs. WT; ^#^ *p* < 0.05 vs. APP/PS1 without BBR.

To determine whether BBR could ameliorate cognitive impairment in APP/PS1 mice, MWM test was performed to appraise learning and memory abilities of mice (Figure 2A). The results revealed that APP/PS1 mice had diminished reference, procedural spatial learning ability (Figures 2B–D) but had increased the escape latency, while APP/PS1+BBR groups showed a gradual narrowing of time slot after training. The results also revealed that APP/PS1 mice spent much shorter time to stay in the target quadrant, and BBR administration obviously increased the time (Figure 2E). However, the swimming distance in all animals remained consistent (Figure 2F).

### Berberine Reduces Amyloid Beta 42 Production and Deposition in Amyloid Precursor Protein/PS1 Mice

**FIGURE 3:**
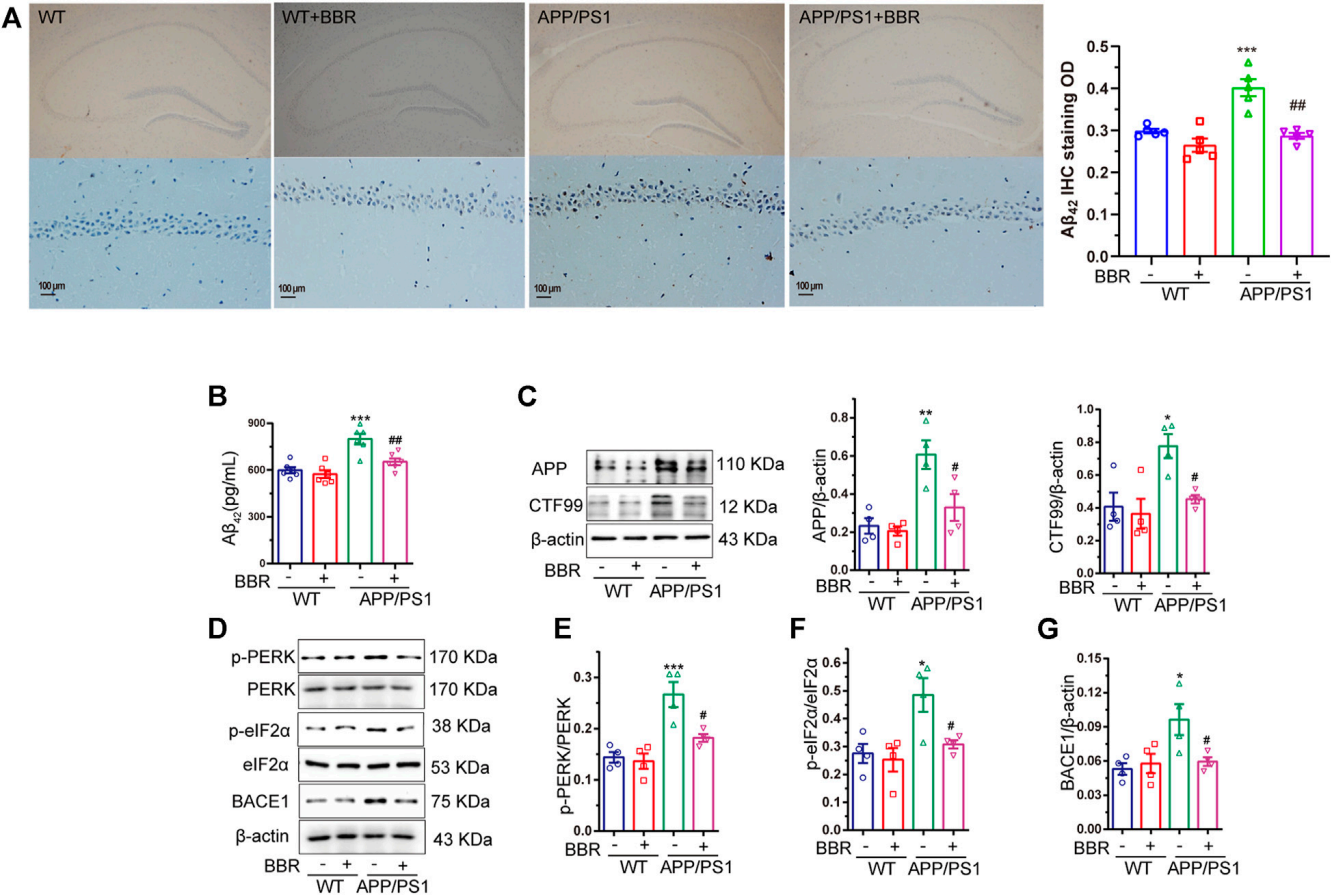
Berberine reduces Aβ_42_ production and deposition in APP/PS1 mice. **(A)** Representative IHC staining images and quantification of Aβ_42_ in the CA1 region of the hippocampal tissue of mice. Scale bar is 100 μm. **(B)** The contents of Aβ_42_ in the supernatant of hippocampal tissue homogenate of APP/PS1 mice were detected by ELISA. **(C)** Western blot detection of APP and CTF99 expression in the hippocampal tissues of the mice, and quantitative analysis. **(D–G)** Western blot detection of p-PERK, PERK, p-eIF2α, eIF2α, and BACE1 in the hippocampal tissues of mice, and quantitative analysis of p-PERK, p-eIF2α, and BACE1. Data were presented as means ± SEM. *p < 0.05, **p < 0.01, ***p < 0.001 vs. WT; ^#^ *p* < 0.05, ^##^ *p* < 0.001 vs. APP/PS1 without BBR.

The amount of Aβ_42_ was detected in the hippocampal tissue of the mice by the IHC assay. The results revealed brown spot that indicated positive signal for Aβ_42_, which was more in APP/PS1 mice when compared with WT mice, while BBR treatment significantly reduced the density of positive signal (Figure 3A). Quantification of Aβ_42_ by ELISA showed similar results (Figure 3B).

It is well known that Aβ_42_ is the cleaved fragment of APP by BACE1, and CTF99 is the byproduct of APP cleavage. Hence, the amount of APP and CTF99 in the mice hippocampal tissue was determined. The results revealed that higher amounts of APP and CTF99 were observed in APP/PS1 mice, but BBR treatment significantly reduced the amount of APP and CTF99 (Figure 3C). This might explain the reason for less production of Aβ_42_.

ER stress could regulate several protein translational regulatory activities including eIF2α (Guan et al., 2014). So, it is hypothesized whether ER stress-activated eIF2α might be related to this process. The results showed that the phosphorylation levels of eIF2α as well as PERK, and the kinase that phosphorylated eIF2α were significantly increased in APP/PS1 mice, which represented its activation, while BBR treatment could effectively reduce its phosphorylation level (Figures 3D–F). Several studies have demonstrated that eIF2α could regulate the expression of BACE1 that could cleave APP to produce Aβ_42_ (O’Connor et al., 2008; Mouton-Liger et al., 2012; Devi and Ohno, 2014). Also, BACE1 was shown to be obviously upregulated in APP/PS1 mice, while returning to normal expression after BBR treatment (Figures 3D,G).

### Berberine Suppresses Glycogen Synthase Kinase 3β Activity and Decreases Tau Phosphorylation

**FIGURE 4:**
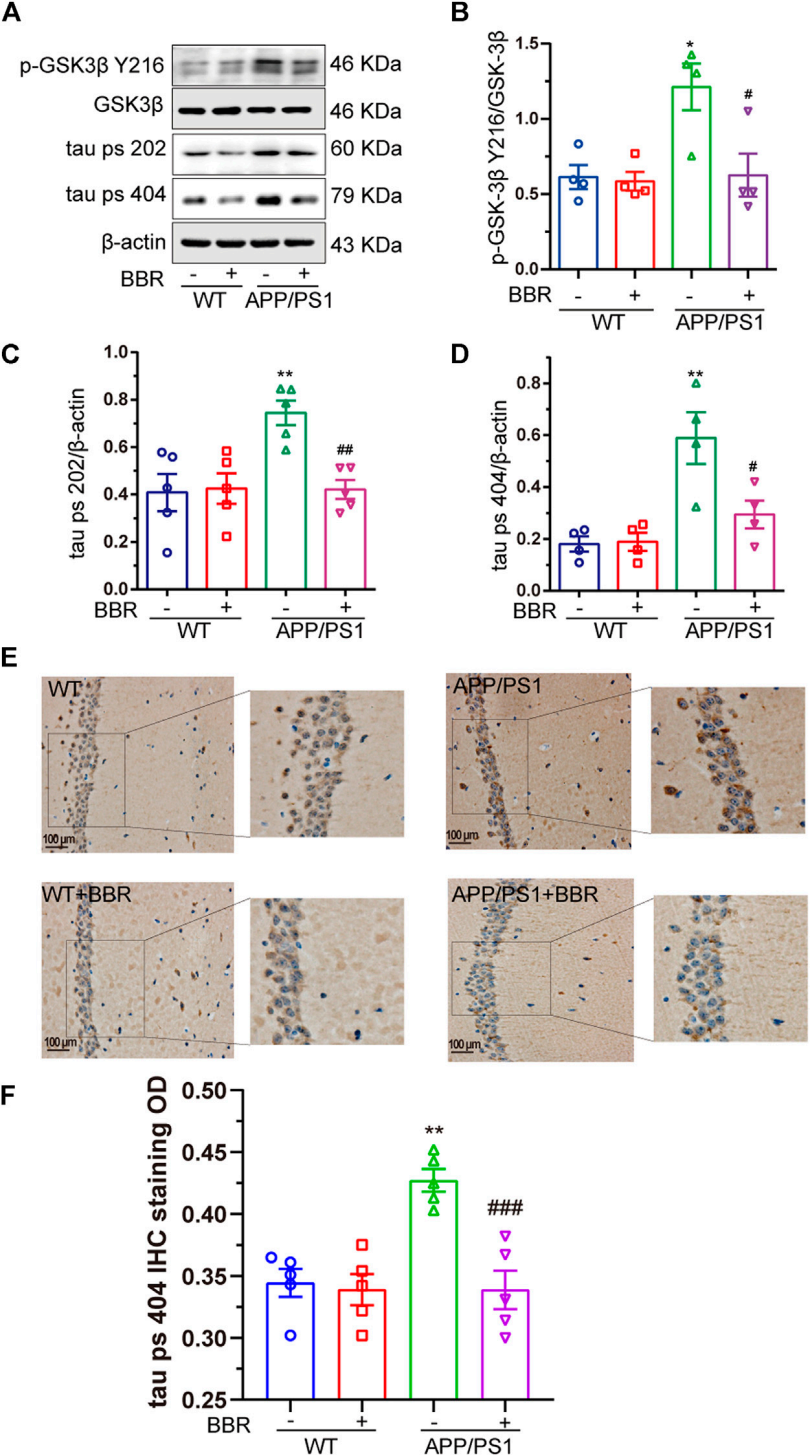
Berberine suppresses GSK3β activity and decreases tau hyperphosphorylation in APP/PS1 mice. **(A)** Western blot detection of p-GSK3β Y216, GSK3β, tau ps 202, and ps 404 in the hippocampal tissues of mice. **(B–D)** Quantification of Western blotting results of p-GSK3β Y216, tau ps 202, and ps 404 in the hippocampal tissues of mice. **(E)** Representative IHC staining images of tau ps 404 in the CA1 region of mice hippocampal tissues. Scale bar is 100 μm. **(F)** Quantification of IHC staining. Data were presented as means ± SEM. *p < 0.05, **p < 0.01 vs. WT; ^#^ *p* < 0.05, ^##^ *p* < 0.01, ^###^ *p* < 0.001 vs. APP/PS1 without BBR.

Another important feature of AD involves the hyperphosphorylation of tau. Several key kinases could phosphorylate tau, and GSK3β among these is thought to be the most important one (Medina et al., 2011). Hence, the activity of GSK3β in the hippocampus was tested. The results showed that the activity of GSK3β was obviously upregulated due to the phosphorylation of GSK3β at Y216 site, which is the active site, showing a significant increase in APP/PS1 mice (Figures 4A,B). As a result, phosphorylation of tau was also significantly increased in APP/PS1 mice (Figures 4A, C, D). It is quite exciting to find that BBR treatment significantly attenuated GSK3β activation as well as tau hyperphosphorylation. Similar results could be found in the mouse hippocampus after using the antibody against phosphorylated tau at 404 site (Figures 4E,F).

Taken together, these data indicated that BBR treatment could significantly ameliorate ER stress in APP/PS1 mice and attenuate two dominant pathological changes of AD, which include the production and deposition of Aβ_42_ and tau hyperphosphorylation.

### Berberine Alleviates Thapsigargin-Induced Endoplasmic Reticulum Stress in HT22/Amyloid Precursor Protein Cells

**FIGURE 5:**
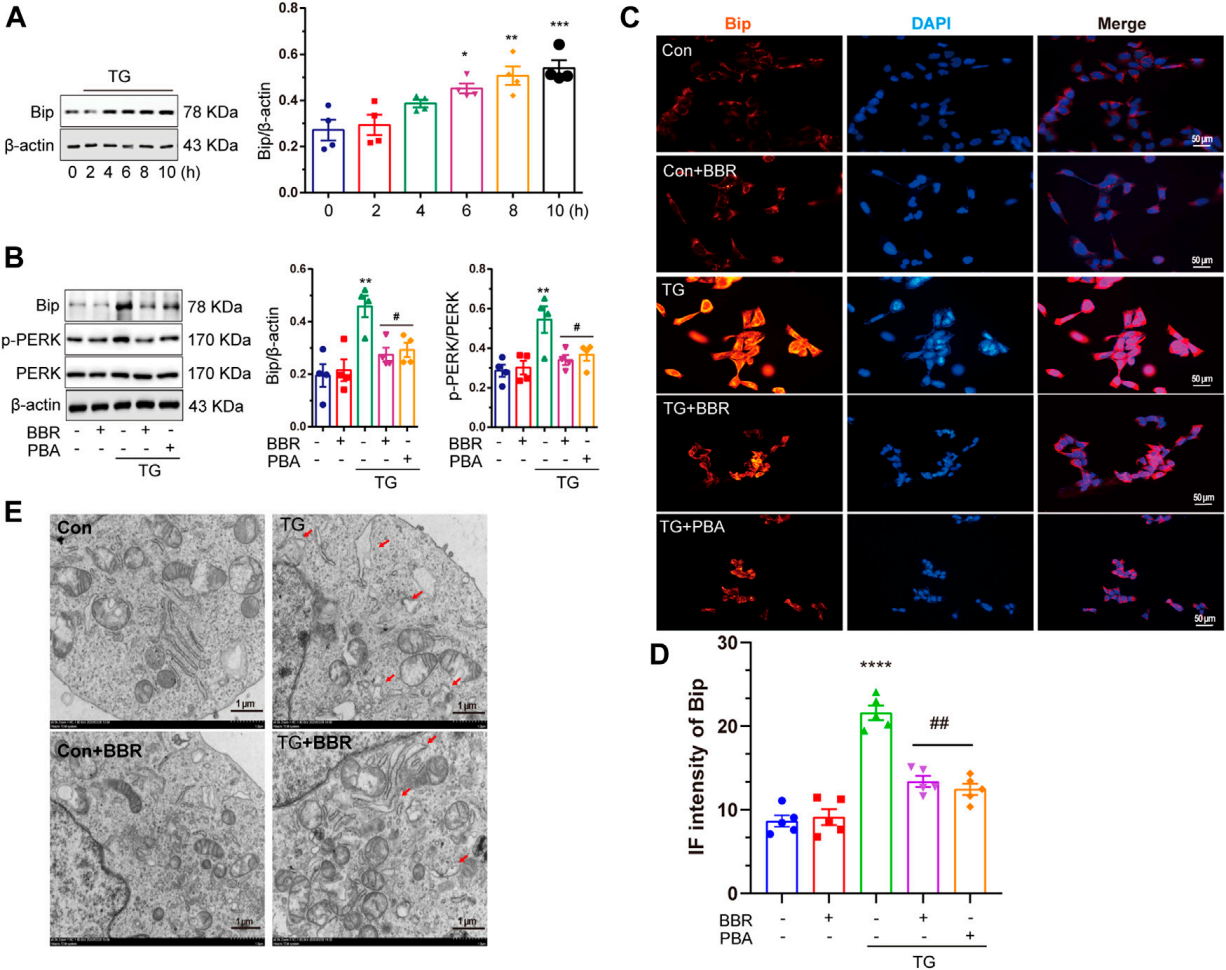
Berberine mitigates ER stress induced by thapsigargin in HT22/APP cells. **(A)** Western blot detection of Bip in TG-induced HT22/APP cells at different treatment time points and quantitative analysis. **(B)** Western blot detection of Bip, p-PERK, and PERK in TG-induced HT22/APP cells with or without PBA or BBR and quantitative analysis. **(C)** Representative immunofluorescence images of Bip in TG-induced HT22/APP cells. Scale bar is 50 μm. **(D)** Quantification of IF of Bip. **(E)** Representative TEM images of the endoplasmic reticulum structure in TG-induced HT22/APP cells. Scale bar is 1 μm. Data were presented as means ± SEM. *p < 0.05, **p < 0.01, ***p < 0.001, ****p < 0.0001 vs. Blank; ^#^ *p* < 0.05, ^##^ *p* < 0.01 vs. TG.

After confirming ER stress in APP/PS1 mice, the HT22/APP cells were used as a cell model for conducting *in vitro* investigations. Thapsigargin (TG) is a classical drug that induces ER stress (Zhang et al., 2014). TG 1μM was selected to treat HT22/APP cells for 8 h to induce ER stress in the mouse cell model after tittering for treatment time period (Figure 5A). Under ER stress, the phosphorylation of PERK was upregulated, while BBR downregulated Bip expression and phosphorylation of PERK, which was similar to the results of PBA, a classical ER stress protector (Figure 5B). Similar results were confirmed by immunofluorescence of Bip in cell model (Figures 5C,D) as well as by TEM (Figure 5E).

### Endoplasmic Reticulum Stress Contributes to Amyloid Beta 42 Production, and Berberine Decreases Beta-Site APP Cleaving Enzyme-1 Expression by Suppressing Endoplasmic Reticulum Stress

**FIGURE 6:**
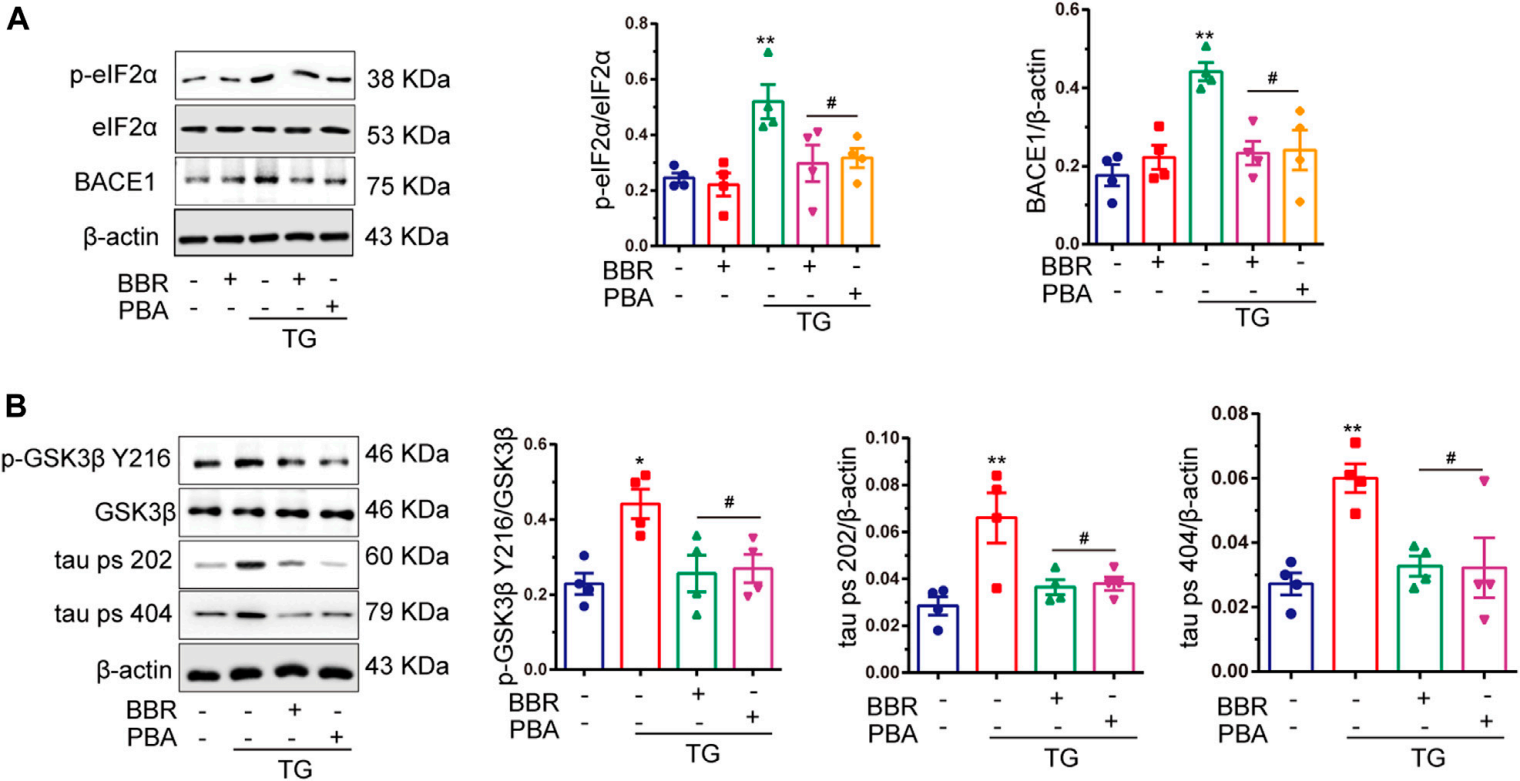
Berberine inhibits tau hyperphosphorylation, as well as BACE1 production and eIF2α activation. **(A)** Western blot detection of p-eIF2α, eIF2α, and BACE1 in TG-induced HT22/APP cells with or without PBA or BBR and quantitative analysis. **(B)** Western blot detection of p-GSK3β Y216, GSK3β, tau ps 202, and tau ps 404 in TG-induced HT22/APP cells with or without PBA or BBR and quantitative analysis. Data were presented as means ± SEM. *p < 0.05, **p < 0.01 vs. Blank; ^#^ *p* < 0.05 vs. TG.

BACE1 is tightly related to the formation of Aβ_42_, and so the expression of BACE1 in HT22/APP cells was detected. The results showed that after treatment with TG 1 μM, the expression of BACE1 was shown to be significantly increased, while BBR as well as PBA interference reversed its upregulation (Figure 6A). As a potential regulator of BACE1, phosphorylation of eIF2α was found to be upregulated in TG stimulated cells, mimicking the *in vivo* results, and hyperphosphorylation of eIF2α was attenuated by BBR and PBA treatment (Figure 6A). These data indicated that BBR plays a role as a ER stress protector like PBA did.

### Endoplasmic Reticulum Stress Contributes to Tau Hyperphosphorylation by Activating Glycogen Synthase Kinase 3β, and Berberine Reverses This Process

The phosphorylation of GSK3β and tau protein was significantly upregulated under TG stimulation (Figure 6B). Interestingly, BBR treatment perfectly reversed tau hyperphosphorylation induced by TG stimulation, which was similar to that of PBA. This indicated that BBR indeed attenuated ER stress caused by TG. In summary, these data strongly supported the idea that BBR could alleviate ER stress in HT22/APP cells, reducing the pathological phenotypes of AD in the cell model.

### Berberine Decreases Amyloid Beta 42 Production by Inhibiting Eukaryotic Translation Initiation Factor-2 α Activation, While Eukaryotic Translation Initiation Factor-2 α Shows Little Effect to Glycogen Synthase Kinase 3β

**FIGURE 7:**
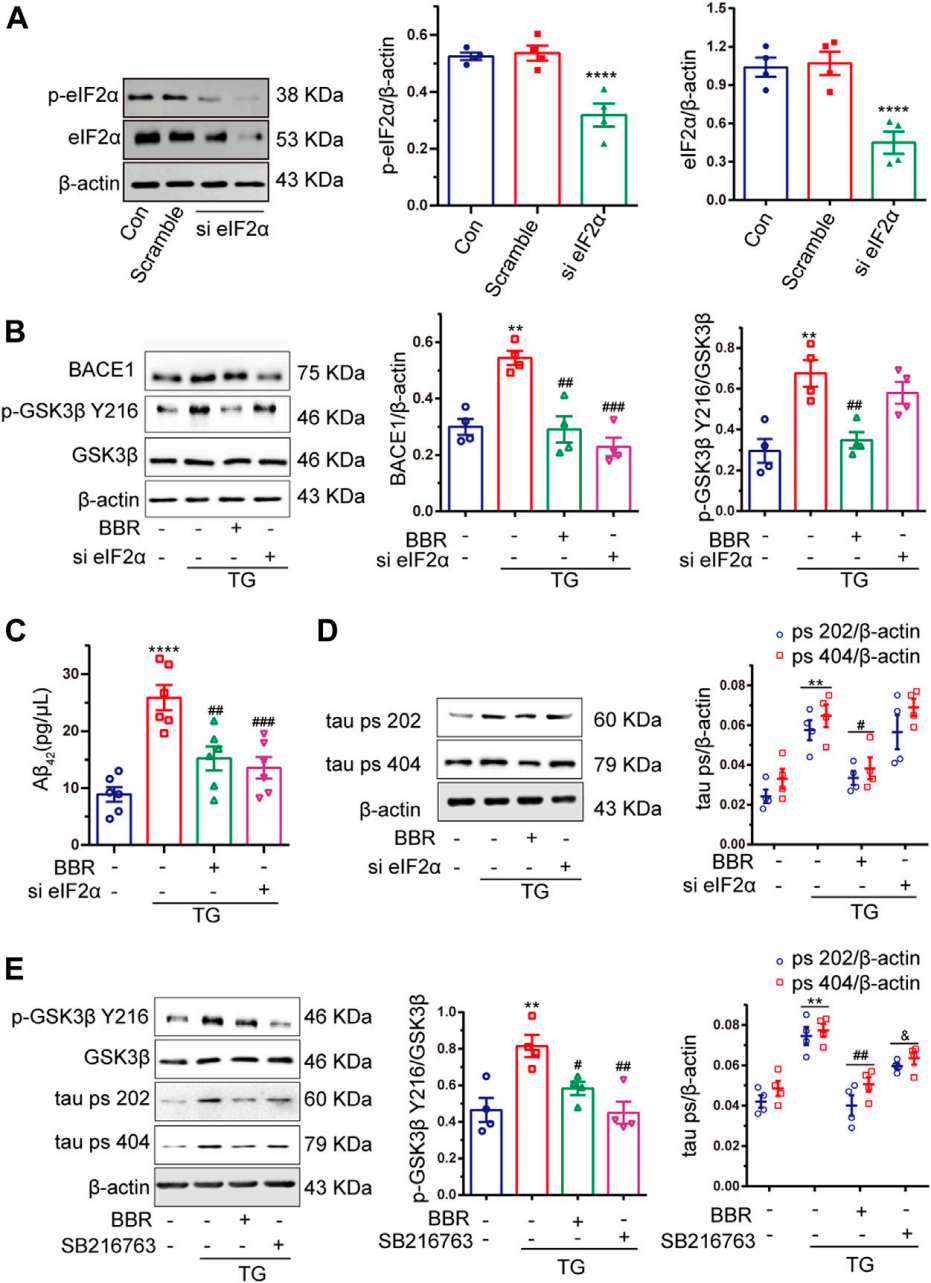
Berberine decreases Aβ_42_ production by inhibiting eIF2α activation, but eIF2α had little effect to GSK3β. **(A)** Western blot detection of eIF2α and p-eIF2α in HT22 cells with si-eIF2α (siRNA eIF2α) or scramble and quantitative analysis. **(B)** Western blot detection of BACE1, p-GSK3β Y216, and GSK3β in TG-induced HT22/APP cells with or without si-eIF2α (siRNA eIF2α) or BBR and quantitative analysis. **(C)** The contents of Aβ_42_ in TG-induced HT22/APP cells with or without si-eIF2α (siRNA eIF2α) or BBR were detected by ELISA. **(D)** Western blot detection of tau ps 202 and tau ps 404 in TG-induced HT22/APP cells with or without si-eIF2α (siRNA eIF2α) or BBR and quantitative analysis. **(E)** Western blot detection of p-GSK3β Y216, GSK3β, tau ps 202, and tau ps 404 in TG-induced HT22/APP cells with or without SB216763 (GSK3β inhibitor) or BBR and quantitative analysis. Data were presented as means ± SEM. *p < 0.05, **p < 0.01, ***p < 0.001, ****p < 0.0001 vs. Blank; ^#^ *p* < 0.05, ^##^ *p* < 0.01, ^###^ *p* < 0.001 vs. TG, ^&^ *p* < 0.05 vs. BBR.

As shown above, BBR attenuates ER stress in the AD model both *in vivo* and *in vitro*. It has been reported that eIF2α can be phosphorylated by activated PERK in the ER stress signal transduction pathway. As a translation regulator, eIF2α might contribute to the regulation of numerous protein biosyntheses (Schroder and Kaufman, 2006). This led us to think whether eIF2α might be the key in regulating ER stress downstream signal transduction. Hence, by using siRNA targeting at eIF2α, the ability to silence eIF2α as well as its activity in HT22/APP cells was confirmed (Figure 7A). After eIF2α was successfully silenced, TG treatment in APP stably expressed HT22 cells were used to mimic ER stress in AD mice, and then investigate the changes on Aβ_42_ and tau phosphorylation. As shown in Figure 7B, si-eIF2α significantly decreased BACE1 expression, while showing little effect to GSK3β activation. BACE1 downregulation decreased the concentration of Aβ_42_, confirming the effect of BBR in reducing Aβ_42_ production (Figure 7C). Although si-eIF2α had great effect in attenuating BACE1 expression and Aβ_42_ production, which showed no effect on tau phosphorylation (Figure 7D). Hence, this indicated that interference with only one pathological process of AD might not be considered useful in treating AD. In contrast, BBR treatment can significantly downregulate BACE1 expression as well as GSK3β phosphorylation (Figure 7E) similar to that of SB216763, which is a specific GSK3β inhibitor. This led to decreased production of Aβ_42_ and tau phosphorylation, showing comprehensive ability in ameliorating ER stress effects in the AD model.

## Discussion

Recently, ER stress is gaining more and more attention in investigating the etiology of AD (Gerakis and Hetz, 2018). In our recent study, evidences with regard to BBR showed alleviation of tau hyperphosphorylation and Aβ_42_ deposition. Most importantly, it has been confirmed that the effect of BBR mainly depended on attenuation of ER stress.

Under pathological stimulation, numerous newly synthesized proteins could not be folded into correct construction, and this might be delayed in the ER lumen, causing unfolded protein response and leading to upregulation of Bip, which is an important molecular chaperone that helps protein folding into correct structure (Yoshida, 2007). In our research, a significant elevation of Bip was found *in vivo* model of AD, which could be perfectly reversed by BBR treatment. This strongly indicated that BBR could effectively attenuate ER stress, as it is a central hub of cellular signal transduction in AD.

As one of the major kinases that phosphorylate tau protein (Hernandez et al., 2012), GSK3β has drawn much attention in the field of AD. Interestingly, several studies have indicated that the activity of GSK3β could be modulated by ER stress (Liu et al., 2017). It is reported that the abnormality in GSK3β activity could trigger numerous intercellular dysfunctions (Nie et al., 2016; Zaouali et al., 2017; Li et al., 2020). Our study provided evidence that the activity of GSK3β was upregulated in the AD model, which in turn resulted in the hyperphosphorylation of tau in the hippocampus. This led us to consider whether the inhibitory effects of tau hyperphosphorylation by BBR in the AD mice model was also related to the inhibition of GSK3β activity. The subsequent data of our study clearly supported the hypothesis. Liu et al. also have reported that the ER stress marker Bip protein could enhance the association of GSK3β with tau protein, which might explain the reason for tau hyperphosphorylation (Liu et al., 2012). In another cell model of AD, in which Aβ was applied to stimulate the cell, the occurrence of ER stress and tau hyperphosphorylation have been found (Resende et al., 2008; Hoozemans et al., 2009). It is worthy to observe that BBR could attenuate Bip expression, which might be the mechanism behind its inhibitory effect to GSK3β.

As the major component of β-secretase, BACE1 is considered responsible for APP cleavage to regulate its appropriate amount (O’Brien and Wong, 2011). However, under several stress stimulations, BACE1 is shown to be upregulated (Penke et al., 2017). The physiological function of BACE1 to cleave APP, in other words, to degrade APP might be responsible for its upregulation to maintain the intracellular homeostasis (Salminen et al., 2013). What turns the situation more worse is that BACE1 tends to cleave APP into pro-oligomerized Aβ_42_ rather than the soluble form of Aβ_40_ (Li et al., 2006). Also, BACE1 expression was also shown to be upregulated, accompanied by elevation of Aβ_42_ in APP/PS1 mice as well as TG-stimulated HT22/APP cells. BBR showed excellent ability in reducing BACE1 expression as well as Aβ_42_ formation both *in vivo* and *in vitro*. This led us to think whether this is related to the alleviation of ER stress.

PERK is activated under ER stress, and then subsequentially phosphorylates the downstream eIF2α and modulates the process of translation initiation (Li et al., 2015). It has been reported that eIF2α could modulate transcription of several key molecules in other diseases, including abdominal aortic aneurysm (Ni et al., 2018), nonalcoholic fatty liver disease (Gao et al., 2016), and drug-induced liver dysfunction (Wang et al., 2016). Indeed, eIF2α was significantly activated under ER stress both *in vivo* and *in vitro*. Also, siRNA was used to downregulate the expression of eIF2α and found a significant decrease in Aβ_42_ production by downregulating BACE1 expression. The results of this study demonstrated that eIF2α was vital for abnormal upregulation of BACE1 and Aβ_42_, as well as for the formation of senile plaques. However, only silencing of eIF2α showed little effect to GSK3β, indicating that different signaling pathways might be involved in the formation of senile plaques and hyperphosphorylated tau. Hence, interference with one of the pathways could obtain limited benefits, and a central signaling hub such as ER stress should be mentioned instead.

In the present study, the evidences that ER stress could be the central signaling hub in the development of AD, and BBR treatment showed excellent protective effects to ER stress, make it possible to be used in the treatment of AD. In fact, BBR is now widely used in the treatment of several diseases (Ji and Shen, 2011; Ruan et al., 2017; Feng et al., 2019). Also, one of the advantages of BBR in treating dementia is that it could travel through the blood–brain barrier, showing the effects to the central nervous system (Kumar et al., 2015). Additionally, BBR shows very low toxicity and gastrointestinal side effects and mildly upset stomach after oral administration (Chen et al., 2014). Taking this advantage, BBR was shown to be effective in inhibiting the hippocampal ER stress occurrence as well as downstream signaling pathways, including GSK3β activation and BACE1 overexpression.

In recent years, too many drugs including BACE1 inhibitor, receptor for advanced glycation end-product inhibitor, and Aβ vaccine have shown good therapeutic potential in early clinical trials, but turn out to be with largely disappointing results. Up-to-date, only two new pharmacological therapies have been licensed for the treatment of AD: memantine and oligomannate; the latter is only licensed in China, but no pharmacological treatments have become available for use in individuals with mild cognitive impairment (Ballard et al., 2020). Although research concerning AD is moving away from the inhibitor development of targeting the traditional senile plaques and NFTs to new monoclonal antibody drugs, such as Gantenerumab (Roche), Solanezumab (Lilly), and Aducanumab (BiogenInc), still have few interesting results. So, enhanced traditional drug repositioning and repurposing may accelerate the identification of new treatments for individuals with AD dementia and mild cognitive impairment (Ballard et al., 2020).

**FIGURE 8:**
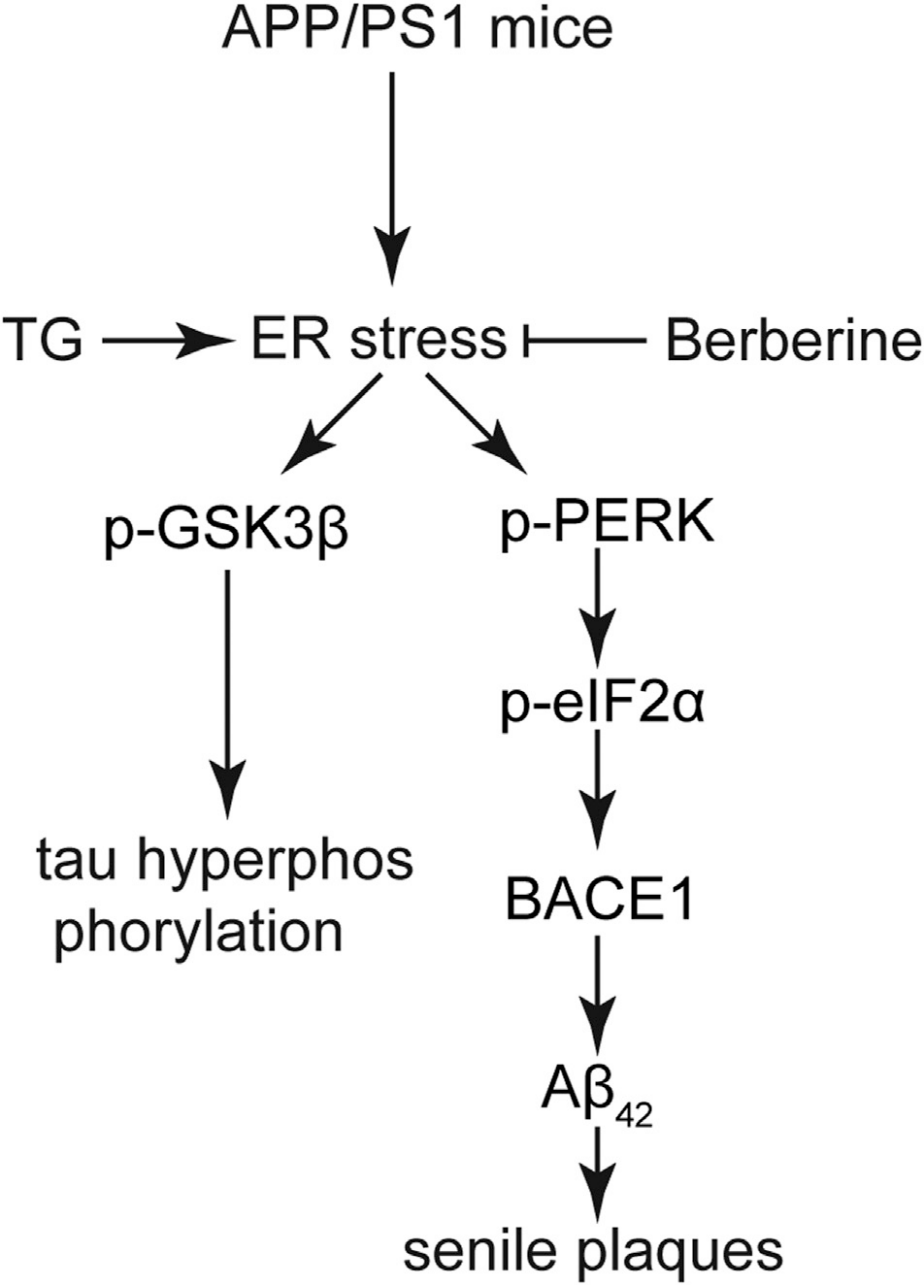
A functional model of berberine-protecting effects against ER stress induced production of senile plaques and hyperphosphorylation of tau in AD. In the APP/PS1 AD model, ER stress was induced, and subsequently activated GSK3β cascade and the PERK/eIF2α/BACE1 signaling pathway. Tau hyperphosphorylation and Aβ_42_ deposition were attenuated by BBR, which is mainly attributed to its ability to suppress ER stress in the AD model.

In conclusion, our work demonstrated that BBR acts as an effective agent in relieving AD both *in vivo* and *in vitro*. The anti-AD effect of BBR relies on the amelioration of ER stress, which can inhibit the overactivation of GSK3β to prevent the hyperphosphorylation of tau, as well as inhibit eIF2α activation to reduce BACE1 expression (Figure 8). Also, the evidence that ER stress acts as the central hub in the etiology of AD, which links the formation of senile plaques and hyperphosphorylation of tau, provides new insights for future drug development in treating AD.

## Data Availability

The original contributions presented in the study are included in the article/Supplementary Material; further inquiries can be directed to the corresponding authors.
